# Stability of Deficits in Reading Fluency and/or Spelling

**DOI:** 10.1080/10888438.2019.1659277

**Published:** 2019-09-02

**Authors:** Kristina Moll, Melanie Gangl, Chiara Banfi, Gerd Schulte-Körne, Karin Landerl

**Affiliations:** a Department of Child and Adolescent Psychiatry, Psychosomatics, and Psychotherapy, Ludwig-Maximilians-University Munich, Munich, Germany; b Institute of Psychology, University of Graz, Austria; c Department of Cognitive Science, Macquarie University, Sydney, NSW

## Abstract

Deficits in reading fluency and in spelling can dissociate during development, resulting in groups with reading deficit only (RD), spelling deficit only (SD) and combined reading and spelling deficit (RSD). The current study investigated the one-to-two-year longitudinal stability of these subgroups in 167 German-speaking children. Reading fluency deficits (irrespective of spelling skills) were stable over time, while spelling deficits were stable in the RSD-group but not in the SD-group. Lower stability in the SD-group resulted from the fact that many children improved their spelling skills over time. Improvement in spelling was associated with good performance in phoneme awareness together with intact RAN and decoding skills.

Inter-individual differences in reading skills are highly stable over time and this stability is largely due to genetic influences (Astrom, Wadsworth, & DeFries, 2007; Harlaar, Dale, & Plomin, 2007; Petrill et al., 2007; Raskind, 2001; Wadsworth, DeFries, Olson, & Willcutt, 2007). Evidence for reading stability is mainly based on samples acquiring the inconsistent English orthography, therefore focusing on reading *accuracy*. However, the few existing studies analyzing the stability of reading *fluency* in consistent orthographies also suggest relatively high stability with correlations of .76 and .64 (Klicpera & Schabmann, 1993; Landerl & Wimmer, 2008). Thus, longitudinal stability has been reported for both reading accuracy and fluency for samples covering the whole distribution of reading skills. Nevertheless, the question remains whether stability is also high for individuals at the bottom end of the distribution, and particularly for reading fluency deficits (cf., Tressoldi, Stella, & Faggella, 2001), which constitute the main criterion for dyslexia in consistent orthographies (Wimmer & Schurz, 2010).

Another question relates to the heterogeneity of dyslexia and the fact that stability might differ according to the literacy components that are affected. For example, longitudinal stability has been found to be stronger for phonological dyslexia (poor pseudoword reading but spared irregular word reading) than for surface dyslexia (poor irregular word reading but spared pseudoword reading) (Manis & Bailey, 2008; Peterson, Pennington, Olson, & Wadsworth, 2014).

The current study focusses on the literacy components of reading fluency and spelling, as prevalence studies report dissociations (Fayol, Zorman, & Lété, 2009; Moll & Landerl, 2009; Wimmer & Mayringer, 2002), resulting in subgroups with isolated reading deficits (RD), isolated spelling deficits (SD), and combined reading and spelling deficits (RSD) (Landerl & Moll, 2010; Moll, Kunze, Neuhoff, Bruder, & Schulte-Körne, 2014; Moll & Landerl, 2009; Torppa, Georgiou, Niemi, Lerkkanen, & Poikkeus, 2017; Wimmer & Mayringer, 2002). These subgroups are associated with different cognitive profiles. While children with RD show more deficits in rapid-automatized naming (RAN) but less marked deficits in phonological awareness, children with SD show the opposite pattern (Furnes, Elwér, Samuelsson, Olson, & Byrne, 2019; Moll & Landerl, 2009; Wimmer & Mayringer, 2002; Wolf & Bowers, 1999).

According to the Lexical Quality Hypothesis, high-quality representations guarantee fluent word reading as well as accurate spelling (Perfetti, 2007; Perfetti & Hart, 2002; Reitsma, 1983) and developmental models proposes a close link between reading and spelling (Ehri, 1997; Perfetti, 2007). The question arises then, how dissociations between reading fluency and spelling can be explained and whether they represent reliable and stable patterns. This is particularly interesting for dysfluent reading in the context of age-adequate spelling, a pattern that has mainly been documented in orthographies with high grapheme-phoneme consistency, which often have clearly lower consistency in the spelling direction (Gangl et al., 2018; Moll & Landerl, 2009; Wimmer & Mayringer, 2002). Spelling is, therefore, a good indicator of a child´s word-specific knowledge and it is surprising that some children with typical spelling development are unable to read fluently.

So far, RD- and SD-groups have only been investigated cross-sectionally and it is possible that dissociations reflect poor performance during particular assessments rather than persisting disorders. In the context of a research project investigating causal mechanisms underlying such dissociations, we had the opportunity to analyze longitudinal stability one or two years later. Three specific research questions (Qs) were addressed:

(Q1) How stable are literacy skills in a relatively consistent orthography (German)?

(Q2) Are there differences in stability of RD-, SD-, and RSD-subtypes of dyslexia?

(Q3) Do subtypes differ in standard cognitive predictors of literacy skills, namely phoneme awareness (PA), rapid automatized naming (RAN), verbal memory, and IQ? Is stability related to cognitive profiles?

## Method

### Participants

German-speaking children with different reading and spelling profiles (mean age 9;5 years) were selected at two collaborating sites (University of Graz, Austria and University of Munich, Germany). In total, 4123 children from 114 schools and 276 classrooms took part in the screening phase, followed by an individual assessment (T1) at the end of Grade 3. All T1-participants were re-invited to a second assessment (T2) 1 year later at the end of Grade 4 (cohort 1: mean age 10;4 years) or two years later at the end of Grade 5 (cohort 2: mean age 11;5 years).

Children were selected based on (1) standardized classroom measures of sentence reading fluency and spelling and on (2) individually administered word and pseudoword reading efficiency subtests. The deficient performance was defined as a percentile at or below 20 in spelling and/or word reading (mean of sentence and word reading subtests as well as across all three reading measures). Scores equal to or above percentile 25 in spelling and/or word reading as well as across all three reading measures indicated age-adequate performance. To select a well-matched control group with typical development, children with exceptionally high performance (above percentile 85) were not invited.

Further inclusion criteria were German as first language, nonverbal IQ not below 85, and no history of neurological disorders. Children with a diagnosis of AD(H)D or attentional problems reported in a standardized questionnaire (Döpfner, Görtz-Dorten, Lehmkuhl, Breuer, & Goletz, 2008) were excluded. The study was approved by the ethics committees of the University of Graz and the University of Munich and was performed in accordance with the latest version of the Declaration of Helsinki and in compliance with national legislation. All parents of children participating in the study gave their written informed consent.

At T1, 203 children participated: 31 RD, 47 SD, 52 RSD, and 73 TD. Of these, 167 could be assessed again at T2 (cohort 1: *n* = 131; cohort 2: *n* = 36). Comparing literacy skills of the 36 children who dropped out (6 RD, 10 SD, 9 RSD, 11 TD) with those who were reassessed using non-parametric Mann-Whitney-U-Tests, showed no evidence for selective attrition in any of the groups (all *p*s > .05). The data reported are based on the 167 children with complete data sets.

## Materials and procedure

### First assessment (T1)

#### Reading fluency

In a standardized classroom reading fluency test ((Wimmer & Mayringer, 2014); parallel-test reliability above .86) children read simple sentences silently and marked them as semantically right or wrong (e.g., “Trees can speak.”). Test score was the number of sentences correctly marked within 3 mins.

In an individually administered 1-min reading fluency test ((Moll & Landerl, 2010); parallel-test reliability between .90 and .95) children read a word and a pseudoword list aloud as fast as possible without making errors. The number of items read correctly was scored for each subtest.

#### Spelling

The standardized classroom spelling test ((Müller, 2004); parallel-test reliability = .92) contained 44 words that were written into sentence frames. The number of incorrect word spellings was scored.

#### Nonverbal IQ

Four subtests of the German version of the Culture Fair Intelligence Test ((Weiß, 2006); reliability = .92) were given as an estimate of nonverbal IQ.

#### Attention rating

Parents answered a standardized questionnaire (Döpfner et al., 2008) containing 20 items with a 4-point rating scale. Children with scores above the clinical cutoff for AD(H)D were excluded.

#### Rapid automatized naming

Children named a matrix of 40 monosyllabic digits arranged in five columns and eight rows as quickly and accurately as possible.

#### Verbal memory

The German Digit Span Task (forward and backward) of the Wechsler Intelligence Scale for Children (Petermann & Petermann, 2011) was given.

#### Phoneme awareness

In a computerized phoneme deletion task (Presentation 16.3; (Neurobehavioral Systems, 2012)) with 25 test trials children had to repeat a pseudoword before pronouncing it without a specified phoneme (e.g., “/fɔlt/without/t/” – /fɔl/). Pseudowords not repeated correctly after three presentations were discarded from analysis (0.9% of all items). Cronbach’s alpha for the T1-sample was .76.

### Second assessment (T2)

#### Reading fluency

The 1-min reading test was given (see T1; (Moll & Landerl, 2010)).

#### Spelling

As standardized spelling tests for German are grade-specific, we had to use different tests across time points and cohorts; all assessed the frequency of misspelled words written to dictation (Cohort 1: (Stock & Schneider, 2008); retest reliability = .89; Cohort 2: (Martinez-Méndez, Schneider, & Hasselhorn, 2015); retest reliability = .95).

## Results

Spearman’s rank correlations were run to examine the stability of reading fluency and spelling across time points (Q1). Next, we examined the stability of specific deficit profiles (Q2) by analyzing how many children in each T1-group fulfilled criteria for the same group at T2. To better understand group differences in stability, we analyzed whether groups differed in their literacy and/or cognitive profiles at T1 using ANOVAs (Q3).

### Association between literacy skills at T1 and T2 (Q1)

Composite reading measures were calculated reflecting children’s mean performance across all reading fluency measures applied at T1 (*r*s = .72 – .86) and T2 (*r* = .84), respectively. Correlation between T1 and T2 performance was remarkably high for reading fluency (*r* = .91, *p* < .001), and high to moderate for spelling (*r* = .78, *p* < .001).

### Group stability (Q2)

**Figure 1.**
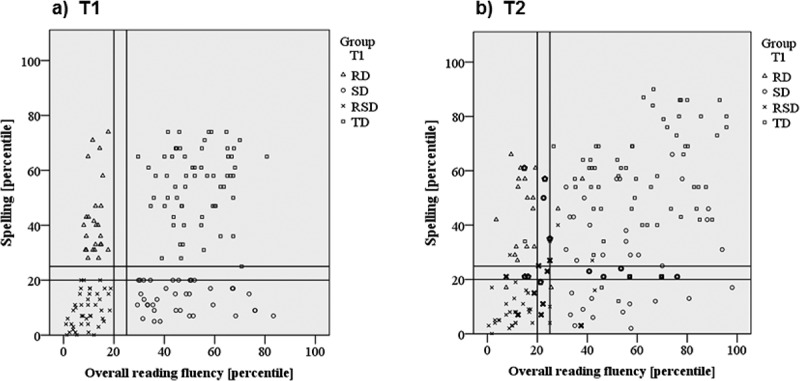
Scatterplot with children’s overall reading fluency (*x*-axis) and spelling performances (*y*-axis) at the (a) first assessment (T1) and (b) second assessment (T2). Overall reading fluency scores indicate children’s mean performance across all reading fluency measures applied at T1 and T2, respectively. Different shapes indicate children’s group affiliation at T1: children with isolated reading deficits (RD), isolated spelling deficits (SD), combined reading and spelling deficits (RSD) and typically developing children (TD). The reference lines at the 20^th^ and 25^th^ percentiles represent the cutoff criteria for group classification. Bold cases in Figure 1b) could not be assigned to any of the groups at T2 either because they scored between the cutoff criteria or because they showed discrepant word and pseudoword reading and therefore did not meet reading criteria (i.e., two children in the RSD- and RD-sections showed a mean reading score below the cutoff but did not meet the deficit criterion for word reading, and one child in the SD-section had a mean reading score in the typical range but did not meet the word reading criterion for typical reading).

Figure 1 presents reading fluency and spelling performance of the four groups at T1 and T2 plotted against each other. Figure 1a mostly reflects the selection criteria. Only a few children performed near the cutoff criteria, while most children in the four groups showed distinctive literacy profiles.

Figure 1b illustrates group stability over time. Most children in the RD-, RSD-, and TD-groups remained in the same group at T2. However, a reasonably high number of SD-children at T1 showed clearly better spelling performance at T2.

Table 1 presents group membership at both time points. At T2, 22 children could not be assigned to a specific group (Figure 1b circled/bold), because they scored between cutoff criteria for reading and/or spelling or showed discrepant word and pseudoword reading.

**Table 1.** Number of children belonging to the four groups at the first (T1) and second assessment (T2).

	Group at T2					
Group at T1	RD	SD	RSD	TD	Not assigned	Total
RD	15	1	1	2	6	25
SD	0	12	0	20	5	37
RSD	1	3	27	3	9	43
TD	1	2	0	57	2	62
Total	17	18	28	82	22	167

RD = children with isolated reading fluency deficits. SD = children with isolated spelling deficits. RSD = children with combined reading fluency and spelling deficits. TD = typically developing children.

Stability did not differ between cohort 1 and 2 (χ^2^ = 0.01, *p* = .91); thus cohorts were combined for further analyses. Although overall stability across groups was reasonably high with 66% falling into the same group at both time points, clear group differences emerged. In line with previous findings (Scarborough, 1998, for an overview), the TD-group was most stable (92% staying in the same group) and stability was significantly higher compared to each deficit group (all χ^2^ > 12.6, *p*s < .001). Stability was moderate in the two reading deficit groups: 60% of the RD and 63% of the RSD children met criteria for the same group at T2. Stability was lowest for the SD-group compared to each of the three other groups (all χ^2^ > 4.7, *p*s < .05): only 32% met criteria at T2. The main reason was that 20 SD-children showed adequate spelling performance at T2.

### Effects of cognitive profiles on stability (Q3)

We analyzed whether (in-)stability in the four groups was related to different literacy or cognitive profiles (Table 2). As the RSD-group was not more impaired than the single deficit groups in T1 reading and spelling, lower stability in the SD-group cannot be explained by less severe spelling deficits compared to the RSD-group.

**Table 2.** Means (standard deviations) for literacy and cognitive measures for the four groups at the first (T1) and second assessment (T2).

	Group at T1							
	RD (n = 25)		SD (n = 37)		RSD (n = 43)		TD (n = 62)	
	T1	T2	T1	T2	T1	T2	T1	T2
Age	114.32 (5.84)	125.60 (5.65)	114.61 (6.45)	128.73 (9.75)	112.70 (5.90)	124.42 (5.85)	112.50 (4.33)	127.21 (7.99)
Reading 1	12.44 (2.80)	16.85 (7.25)	47.21 (15.23)	53.96 (19.84)	9.22 (4.91)	15.16 (10.16)	52.92 (11.19)	62.40 (18.77)
Spelling 1	42.38 (13.99)	41.88 (16.16)	13.32 (5.12)	29.27 (17.67)	9.07 (6.15)	11.79 (9.42)	54.89 (13.08)	58.58 (18.49)
NVIQ	112.32 (14.29)	-	104.19 (10.25)	-	107.02 (12.93)	-	108.10 (10.44)	-
RAN 2	1.81 (0.27)	-	2.01 (0.40)	-	1.78 (0.32)	-	2.17 (0.41)	-
Memory 3	10.96 (2.51)	-	9.81 (2.37)	-	9.58 (2.35)	-	10.45 (2.31)	-
PA 4	75.44 (13.17)	-	75.57 (13.63)	-	63.47 (18.75)	-	81.95 (12.59)	-

^1 ^Percentiles; ^2^ items correct/sec; ^3^ standard-scaled score (M = 10, SD = 3); ^4^ percentage correct.

NVIQ = Nonverbal IQ; RAN = rapid automatized naming of digits; Memory = Verbal memory (digit span); PA = phoneme awareness.

No group differences were found for age and verbal memory; IQ was somewhat higher in the RD-group. In line with previous research (Moll & Landerl, 2009; Wimmer & Mayringer, 2002), deficits in RAN were related to reading problems only. With respect to PA, only the RSD-group performed significantly lower than TD children (*p* < .001) and also lower than the two isolated deficit groups (*p* < .01).

To better understand the instability in the SD-group, we compared the cognitive profiles of SD-children who met criteria for the SD-group at T2 (persistent group) with those who resolved their spelling problems (resolving group). The two groups did not differ in RAN, memory, and IQ (*t*s(35) < 1.4, *p*s > .17), but the persistent group scored lower in PA than the resolving group (67% vs. 80%; *t*(35) = 3.07, *p* = .004), which did not differ from the control group (80% vs. 82%; *p*= .466).^1^

## Discussion

Dyslexia is a heterogeneous disorder and affected individuals differ in symptomatology as well as underlying deficit profiles. Examining if these dissociations are stable over time is of theoretical and practical importance. Here, we assessed the longitudinal stability of dissociations between deficits in reading fluency and spelling.

Most importantly, we found moderate stability for reading fluency deficits, irrespective of spelling performance. In particular, the stability of RD has not been demonstrated before. For spelling deficits, a more complex picture emerged. Spelling problems were moderately stable for children who also had reading fluency deficits (RSD-group), but less stable for children whose reading was intact (SD-group). Next, we discuss potential causal mechanisms and why stability might differ between groups.

### Combined deficits in reading and spelling

In line with findings from English studies suggesting that dyslexia is highly persistent (Astrom et al., 2007; Maughan, Hagell, Rutter, & Yule, 1994; Shaywitz et al., 1999; Wadsworth et al., 2007), we found stability for combined literacy deficits (RSD) in a more consistent orthography. Thus, the stability of RSD does not seem to be related to the definition of the reading deficit (accuracy vs. fluency). On the cognitive level, children with RSD showed a deficit in RAN and in PA, representing the double-deficit profile described by Wolf and Bowers (1999). As a consequence, they are unlikely to overcome their deficit in building-up high-quality orthographic representations, which might explain the high stability observed in this group.

### Reading fluency deficits

Extending previous research based on random samples showing that reading fluency skills are highly stable (Klicpera & Schabmann, 1993; Landerl & Wimmer, 2008), we also found stability for the bottom end of the distribution in both groups with reading deficits (RD and RSD). The fact that children with RD show marked and stable reading deficits despite unimpaired spelling is quite unexpected, given that age-appropriate spelling indicates intact orthographic representations. According to the Lexical Quality Hypothesis (Perfetti, 2007), one would predict that intact representations do not only guarantee accurate spelling but also foster fluent reading. Indeed, recent evidence shows that training spelling improves reading fluency (Ouellette, Martin-Chang, & Rossi, 2017; Rossi, Martin-Chang, & Ouellette, 2019). A plausible explanation for dissociations could be that standardized tests assess reading and spelling skills based on different word-material. However, previous research confirmed dissociations even when the same words were read and spelt (Moll & Landerl, 2009). This raises the question as to why children with RD show problems in reading fluency despite accurate orthographic representations.

We have argued elsewhere (e.g., Bakos, Landerl, Bartling, Schulte-Körne, & Moll, 2018; Moll & Landerl, 2009), that children with RD store and use orthographic representations during reading, but are slow in processing their phonological form. Thus, reading fluency deficits may result from slow visual-verbal access (Lervåg & Hulme, 2009) and inefficient sequential processing (Jones, Obregón, Kelly, & Branigan, 2008; Protopapas, Katopodi, Altani, & Georgiou, 2018; van Den Boer, Georgiou, & de Jong, 2016). This interpretation is supported by strong associations between (deficits in) reading fluency and RAN (Araújo & Faísca, 2019; Araújo, Reis, Petersson, & Faísca, 2015; Kirby, Georgiou, Martinussen, & Parrila, 2010). Given that word reading and RAN both require sequential processing and visual-verbal access, deficits in these processes are likely to explain the RAN-reading relationship. Following this idea, training studies attempted to improve these skills by training RAN (de Jong & Vrielink, 2004) or sublexical units (Huemer, Aro, Landerl, & Lyytinen, 2010; Thaler, Ebner, Wimmer, & Landerl, 2004) with limited training effects. Thus, sequential processing and visual-verbal access seem to be hard to improve which in turn might explain the high stability of reading fluency deficits.

### Spelling deficits

For spelling, we found reasonably high correlations between T1 and T2, but findings were less clear for the bottom end of the distribution. Deficits in spelling were only stable when accompanied by poor reading, while stability was lower when reading was intact.

There are a number of potential methodological reasons for the higher stability for reading than spelling: First, poor reading was defined based on several and mostly the same reading tests across time points, while poor spelling was defined based on one test only which differed across time points. Although the reliability of standardized spelling tests is generally high (current study: .89 and .96), this procedure may have induced incorrect classification of some SD-children who, for some reason, have not performed at their typical spelling level at T1. However, methodological concerns are unlikely to fully account for the low stability of spelling deficits in the SD-group, as stability was low in this group only, but high in the RSD-group^2^. Furthermore, standardized spelling scores did not differ between time points in the two groups of typical spellers, suggesting that the T2-spelling-test was not generally easier than the T1-spelling-test.

A possible explanation relates to the finding that improvement in spelling in the SD-group was related to better PA-performance. Given the reciprocal relationship between PA and literacy skills (Hogan, Catts, & Little, 2005; Landerl et al., 2018; Perfetti, Beck, Bell, & Hughes, 1987), better PA in the context of good decoding skills is likely to foster the buildup of word-specific orthographic representations via self-teaching (Share 1995). We assume that the combination of reasonably good phonological skills, intact RAN together with age-appropriate decoding skills and therefore larger print exposure (Georgiou et al., 2019) may be a protective factor, explaining why SD-children are more likely than RSD-children to overcome early spelling problems.

### Implications and conclusions

The longitudinal stability of RD, SD, and RSD and the observed differences in cognitive profiles provide reasonable evidence for the distinction between reading fluency and spelling deficits and should be considered in educational and clinical practice. The distinction between reading and spelling deficits is directly implemented in the ICD-11 (World Health Organization, 2018). In DSM-5 (American Psychiatric Association, 2013) the distinction can be incorporated by adding specifiers to the overall diagnosis of “learning disorders”.

With respect to prognosis, findings indicate that deficits in reading fluency are highly stable and hard to remediate. Although reading fluency can be improved (Huemer, Landerl, Aro, & Lyytinen, 2008; Thaler et al., 2004), effect sizes are only small (Ise, Engel, & Schulte-Körne, 2012). Future training studies focusing on increasing reading fluency are needed, especially for children who are specifically impaired in this central literacy component. For deficits in spelling, the current findings suggest that they are more likely to be overcome in the context of good reading together with intact naming speed and phonological skills.

Limitations and implications for future research: First, future studies will have to investigate, whether dissociations continue to be stable over a longer follow-up interval. Secondly, cognitive measures were assessed only once, so that developmental changes in cognitive skills and their relation to literacy skills could not be examined. Finally, it is not clear whether the current results can be transferred to deep orthographies. It is quite conceivable that in deep orthographies overcoming spelling deficits via reading practice is harder because decoding may not be unaffected (Frith, 1980). Given that intact decoding is a protective factor to overcome spelling problems, poor spellers in deep orthographies might be more likely to continue to encounter spelling difficulties than children in consistent orthographies.

## Notes

1. Improvement in the SD-group was not related to intervention between assessments: The percentage of SD-children receiving intervention was not higher than in the other deficit groups (SD = 24%; RD = 32%; RSD = 54%).

2. A mock analysis was run to estimate the regression-to-the-mean effect which might explain instability in the SD-group. We simulated the spelling situation by defining reading problems using one reading measure only and a different measure at T1 and T2. Stability was still high in the RD- and RSD-group (65% and 58%).
